# Harmful Alcohol Use

**Published:** 2003

**Authors:** Gerhard Gmel, Jürgen Rehm

**Affiliations:** Gerhard Gmel, Ph.D., is co-director of research at the Swiss Institute for the Prevention of Alcohol and Drug Problems, Lausanne, Switzerland. Jürgen Rehm, Ph.D. is chief executive officer and director of the Addiction Research Institute, Zurich, Switzerland; professor in the Department of Public Health Sciences, University of Toronto; and senior scientist at the Centre for Addiction and Mental Health, Toronto, Canada

**Keywords:** AOD (alcohol and other drug) associated consequences, work-related AOD issue, employee absenteeism, labor productivity, job performance, AODR (alcohol and other drug related) injury, aggressive behavior, AODR violence, AODR crime, AODR family problems, spouse abuse

## Abstract

Alcohol misuse can harm people other than the drinker, and can have negative consequences for society as a whole. It is commonly believed to play a role in decreased worker productivity, increased unintentional injuries, aggression and violence against others, and child and spouse abuse. Research findings support the idea that drinking is involved in or associated with many of these social harms, but do not offer evidence that it causes these effects. Methodological flaws characterize much of the research in this area. Use of better design and statistical methodology is necessary in order to clarify the relationship between drinking and the harmful consequences it is believed to cause.

Alcohol misuse is linked to many harmful consequences for society as a whole and for others in the drinker’s environment. Sometimes referred to as the social consequences of alcohol use (Osterberg 1996; Klingemann and Gmel 2001; Rehm 2001), these negative outcomes are reflected in the diagnostic criteria of alcohol abuse given in the *Diagnostic and Statistical Manual of Mental Disorders, Fourth Edition* (DSM–IV) (American Psychiatric Association [APA] 1994). The DSM–IV defines alcohol abuse as alcohol use that results in:

1. Failure to fulfill major role obligations at work, school, or home (e.g., repeated absences or poor work performance, neglect of children or household)
2. Continued drinking even in situations where it is physically hazardous (e.g., driving an automobile or operating machinery)
3. Recurrent alcohol-related legal problems (e.g., arrests for disorderly conduct while drinking)
4. Continued drinking despite persistent or recurrent social or interpersonal problems it may cause (e.g., arguments with spouse, physical fights).

Reflecting these criteria, this article examines a specific negative consequence from each category, discussing research findings on alcohol use and its relation to workplace absenteeism (criterion 1), unintentional injuries (criterion 2), aggression and violence (criterion 3), and spouse abuse (criterion 4).

Some of these consequences might appear to affect only the drinker; for example, unintentional injuries such as falls often involve only the person who has been drinking. Ultimately, however, these events have an impact on society as a whole insofar as they affect economic productivity or require the attention and resources of the criminal justice or health care systems, or of other social institutions. A review of the research on each of these specific harms is followed by an examination of the methodological issues involved in investigating the consequences of alcohol use.

## Workplace Productivity

In 1998, alcohol abuse and dependence cost the United States an estimated $97.7 billion, primarily as a result of economic productivity lost because of alcohol-related illness, injury, and crime (Harwood 2000, based on Harwood et al. 1998). (This figure does not include loss of future earnings caused by premature death related to alcohol use.) Whether people are in alcoholism treatment, in jail for alcohol-related crimes, or in the hospital as the result of alcohol-related injuries or violence, their incapacity represents a loss in workplace productivity.

Investigators commonly examine workplace injuries, absenteeism, job performance, and turnover when evaluating the effect of alcohol consumption on productivity. In general, research has found that—although moderate consumption may have a beneficial effect on productivity—alcohol dependence, alcohol abuse, and heavy drinking lower productivity. Mullahy and Sindelar (1998) and Sindelar (1998) provide excellent reviews of these studies.

The following discussion focuses on the relationship between alcohol consumption and absenteeism, followed by remarks on job performance.

### Absenteeism

Studies analyzing absenteeism rates of people at all levels of alcohol consumption^1^ have yielded mixed results. Some have found no association between absenteeism and drinking. For example, Ames and colleagues (1997) found no significant association between absenteeism and the drinker’s usual volume of consumption or frequency of heavy drinking occasions (which they defined as occasions during the past year when a person had 10 or more drinks). Moreover, though drinking at the workplace and hangovers at work were related to other negative consequences, such as workplace injuries, they were not related to absenteeism. Other studies, such as that of Blum and colleagues (1993), showed lower absence rates among heavy drinkers than among light drinkers.

Yet other investigators have found that the relationship between drinking and absenteeism could be described by a U-shaped curve—that is, moderate drinkers were absent from work least frequently, but both heavy drinkers and people who drank little were absent more often. A longitudinal study by Marmot and colleagues (1993) found a U-shaped association, for men, between sickness absence and both the frequency of heavy drinking occasions and the weekly quantity consumed. In other words, drinkers whose frequency and quantity of consumption were moderate were absent less often than either abstainers or heavy drinkers. These results are consistent with a large body of evidence demonstrating the existence of such a U-shaped curve for the association between alcohol consumption and heart disease, as well as overall mortality (National Institute on Alcohol Abuse and Alcoholism [NIAAA] 2000). For women, these researchers found no U-shaped association, although abstainers had higher rates of sickness absences than moderate or heavy drinkers.

Few researchers who have examined the whole range of consumption, not just problem drinking, have found a clear correlation between sickness absences and drinking. Jones and colleagues, in a 1995 New Zealand study, found that the workers with the lowest consumption rate had the fewest absentee days, and those with the highest consumption had the highest absence rate. However, interviewers for this study asked questions that directly attributed absences to alcohol consumption: “How many times in the last 12 months have you been away from work because of your drinking?” Wording the question in this way violates standard epidemiological principles of independent measurement of cause and effect and may lead to an overestimation of associations (Gmel et al. 2000). Such questioning may have caused respondents in this study to overstate how much they drank or how much their behavior could be attributed to their alcohol use.

### Job Performance

Various rationales have been put forward to explain why recent research has not shown a correlation between alcohol consumption and absenteeism. Some investigators have suggested that people who are becoming problem drinkers come to work regularly in order to cover up or deny their drinking problem (Trice and Roman 1972) and, possibly for such practical reasons as to avoid using up sick leave or being fired. This strategy could be revealed by lengthy breaks, sleeping on the job, or other measures of low performance. Although there is evidence to support this explanation, it comes from studies in which, again, respondents were asked about their job performance with attribution to their alcohol consumption. One recent study conducted at 114 work sites (Mangione et al. 1999) showed an almost linear relationship between increasing average consumption and a summary measure of job performance, finding the strongest associations between consumption and getting to work late, leaving early, and doing less work, and only a weak association with missing days of work. Thus, alcohol consumption may have more effect on productivity on the job than on the number of workdays missed.

Although using questions that attribute consequences to alcohol use may overestimate the association between drinking and workplace consequences, using participants’ self-reports could underestimate this association. For example, Blum and colleagues (1993) found a stronger relationship between alcohol consumption and work performance when they used reports of coworkers or supervisors instead of relying on self-reported data. However, like self-reports, collateral reports (Sobell et al. 1997) are subject to many sources of error, such as subjectivity and poor recall. Because of the nature of retrospective studies, a large proportion of epidemiologic research has been based on self-reported data. Future research in this area should incorporate objective indicators of work performance, measured independently of exposure to alcohol.

### Confounding Variables

Many factors other than drinking affect work performance in general and absenteeism in particular. Shift work, boredom on the job, repetitive tasks, and workload—just to mention a few—are all related to stress, poor work performance, and drinking (Alberta Alcohol and Drug Abuse Commission 1992; Ames and Janes 1992). This raises a question of reversed causality—that is, whether other workplace factors, such as job-related stress, lead to increased alcohol use as a coping device. The worker’s perception of stress may be more important than stressful working conditions themselves. Vasse and colleagues (1998) showed that work conditions were associated with perceived stress and stress with increased drinking, but that only the combination of work conditions and perceived stress was associated with more absence from work. Among workers who were aware of job-related stress, abstainers had significantly more absences than drinkers. Among workers who did not perceive stress, there was no relation between sickness-related absences and alcohol consumption.

Chains of events not accounted for in experimental design may also cloud the picture of causality. For example, alcohol problems in youth may lead to bad jobs and bad jobs may result in higher absenteeism (see, for example, Kenkel and Wang 1999). Finally, factors such as poor economic conditions or psychiatric disorders may influence both alcohol and work-related outcomes.

## Unintentional Injuries

Studies have shown a high level of alcohol involvement in all types of unintentional injuries (Hingson and Howland 1993; English et al. 1995; Ridolfo and Stevenson 2001). In a meta-analytic review, Smith and colleagues (1999) estimated the percentages of fatal injuries in the United States in 1999 in which alcohol was involved, by type of injury. They found that 38.5 percent of non-traffic-related unintentional injury deaths involved people who had a positive blood alcohol concentration (BAC) (i.e., > 0 mg/dL) and 31 percent involved people who were intoxicated (BAC of ≥ 100 mg/dL) (see table 1). For the largest category of unintentional injury deaths, motor vehicle collision fatalities,^2^ Smith and colleagues found that 39.7 percent of traffic fatalities involved positive BACs, and 32.8 percent involved BACs high enough to indicate intoxication. Though the figures for positive BACs and BACs high enough to indicate intoxication were not directly comparable (see footnote to table 1), they clearly indicate that the involvement of alcohol use in unintentional injuries is high. (The subject of alcohol use and motor vehicle crashes is covered in detail in the articles by Hingson and Winter and by Rehm and colleagues in this issue.) Whether or not unintentional injuries result in death, they have an economic impact in that they can lead to costs for medical treatment, care for the disabled, and decreased or lost workplace productivity (NIAAA 2000).

The fact that alcohol was involved in some way in these injuries does not mean that drinking caused them, but cumulative findings from different types of studies indicate that alcohol plays a causal role. Experimental studies (for reviews, see Moskowitz and Robinson 1988; Krüger et al. 1993; Eckhardt et al. 1998) have demonstrated that even alcohol consumption levels that produce BACs of around 0.05 percent result in impairments of cognitive and psychomotor skills that increase the risk of injury. This is of particular importance because many more people drink at relatively low levels than drink at heavy or problem levels. Emergency Room (ER) studies have consistently shown that injured patients more often have higher BACs than control groups of uninjured people (Cherpitel 1993; Romelsjö 1995). Though BAC readings may be biased,^3^ self-reports of alcohol consumed before the event were also higher in injured ER patients than in uninjured control subjects.

Some evidence indicates that the quantity of alcohol consumed on a given occasion, rather than the person’s usual frequency or volume of drinking, is a powerful predictor of injuries. General population surveys have shown that a greater likelihood of injuries is associated with a drinking pattern in which a person alternates between periods of little or no consumption and episodes of heavy drinking (Gruenewald and Nephew 1994; Gruenewald et al. 1996; Treno and Holder 1997; Treno et al. 1997). At the aggregate level, studies in countries where heavy drinking patterns are relatively common have found higher associations of alcohol consumption not only with unintentional injuries of all kinds, but also specifically with falls (Skog 2001a, b).

### Alcohol Consumption and Different Types of Injury

Some differences between categories of unintentional injury should be noted. For example, alcohol consumption appears to be involved less often with occupational injuries than with other injuries (Webb et al. 1994; Zwerling 1993). Possible explanations for this are (1) intoxicated people may stay away from work in order to hide their drinking; (2) drinking on weekends may give people time to recover before returning to work; or (3) problem drinkers and their supervisors may modify work demands or situations to reduce the likelihood of on-the-job injury (Mangione et al. 1999). However, Dawson (1994), using data from the National Health Interview Survey, showed that the risk of occupational injuries increased with the frequency of heavy drinking (five or more drinks on an occasion). Recently, Zwerling and colleagues (1996) found that older workers who typically drank one to two drinks per day had the lowest risk of injuries (even lower than the risk faced by people who drank less than one drink per day), whereas people who drank five or more drinks a day had a more than fivefold increase in risk.

Injuries as a result of falls are the second most common cause of unintentional fatality (Hoyert et al. 2001). In reviewing data on falls, Hingson and Howland (1993) found that the people involved had consumed alcohol in 18 to 77 percent of the incidents. One of the rare case control studies on falls (Honkanen et al. 1983) suggests a causal role for alcohol, finding that 53 percent of those injured tested positive for alcohol, compared with 15 percent of an uninjured control group. The role of alcohol in falls, however, varies by age, with approximately 30 percent of falls occurring in the oldest 1 percent of the population (i.e., age 85 and over), where alcohol consumption is lowest (Ridolfo and Stevenson 2001). Although few in this age range drink, those who do are more susceptible to alcohol-induced injury, and their injuries may be more serious (NIAAA 1998).

## Aggression and Violence

In the literature, terms such as aggression and violence are often used interchangeably. Aggression, the more inclusive term, is defined as acting with the intention of inflicting some form of harm on others. Aggression can be verbal or indirect (e.g., social exclusion), and can include damage to property (i.e., vandalism) (Björkqvist 1994). Violence is defined more specifically as intentional physical aggression by one person against another (thereby excluding suicide), which can result in serious injury or discomfort. Most alcohol-related offenses are crimes of violence, such as aggravated assault and homicide (Murdoch et al. 1990). (See table 2 for percentages of violent deaths in 1999 that were associated with positive or intoxicating BAC levels.)

Evidence of the link between alcohol and violence comes from experimental and observational studies at both the individual and aggregate levels. This research has not yet answered the fundamental question of whether alcohol is causally related to aggressive behavior (Gelles and Loseke 1993; Lipsey et al. 1997; Pernanen 2001).

### Hypotheses and Experimental Data on Drinking and Aggression

Several theories attempt to explain how alcohol consumption might increase aggression. According to dis-inhibition theory, people’s aggressive tendencies are normally controlled by inhibiting forces. Alcohol would then increase the likelihood of aggressive behavior chemically, through direct pharmacological effects on the brain (Gustafson 1994). However, although alcohol has been shown to affect parts of the brain involved in decisionmaking and impulse control, experimental studies do not support the hypothesis that alcohol’s pharmacological effects alone increase aggressive acts (Bushman 1997).

Social learning theory suggests that alcohol increases aggression because people expect it to do so. The association of alcohol intoxication with aggression would thus be a product of social learning and cultural influences (MacAndrew and Edgerton 1969; Bandura 1973; Lang and Stritzke 1993). In support of this theory, studies show that people act aggressively even if they only believe they have consumed alcohol, as shown by experiments that used placebos (Bushman and Cooper 1990; Gustafson 1994; Bushman 1997; Lipsey et al. 1997). Thus, it appears that pharmacological and expectancy effects may interact to encourage aggressive behavior, although even in combination they are not sufficient to cause aggressive behavior in the absence of other factors (Bushman 1997).

Other theories on drinking and aggression postulate that alcohol contributes indirectly to increased aggression by causing cognitive, emotional, and psychological changes that may reduce self-awareness or result in inaccurate assessment of risks (Bushman 1997). Gustafson (1994) concluded that the experimental data are most consistent with the attentional hypothesis: Drinking constricts an intoxicated person’s attention span, so he or she directs attention to the most salient behavioral stimuli (i.e., cues). If cues that provoke aggression are more noticeable than cues that inhibit it, the person’s subjective feeling of provocation will be increased and hence he or she will behave more aggressively (Gustafson 1994; Lipsey et al. 1997). Alternatively, aggression will decrease if the influence of inhibitory cues such as social norms and social pressure predominates.

The attentional hypothesis has much in common with the selective disinhibition theory developed by Parker and colleagues (Parker 1993; Parker and Rebhun 1995; Parker and Auerhahn 1998). According to this theory, alcohol’s effect on behavior is strongly influenced by the social and cultural context in which it is consumed. For example, in “wet” drinking cultures, alcohol consumption is an almost daily activity with few restrictions on availability. Conversely, in “dry” cultures, legal and social restrictions govern drinking, but binge drinking and even violent drunken behavior are seen as acceptable in some circumstances (Room 2001). Similarly, the social context (e.g., the neighborhood) or the social group (e.g., a party of friends) may inhibit or disinhibit drinking and violent acts (Parker 1993). Parker and Auerhahn (1998) argue that in potentially violent situations a conscious, proactive effort is needed to solve disputes nonviolently, but people may be less likely to make this effort in contexts where violence is more accepted as drunken behavior, such as bars (Graham and West 2001). Although the attentional hypothesis and selective disinhibition theory offer a theoretical and conceptual base for answering questions of causality, they have not yet undergone definitive empirical tests.

### Observational Studies on Violent Crime

According to the National Crime Victimization Survey (NCVS), conducted in the United States between 1992 and 1995 (Greenfield 1998), 37 percent of crime victims reported that alcohol was involved when the crimes against them were committed. Studies using police reports, court documents, or surveys of convicted offenders have found alcohol to be involved in 30 to 90 percent of violent crime. According to Bureau of Justice Statistics (BJS) surveys of the U.S. offender population, about 40 percent of offenders report that alcohol was a factor in their violent offenses (Greenfield 1998). Moreover, the mean BAC estimated from the reports of offenders convicted of violent crimes (homicide, sexual assault, robbery, assault) ranged between 0.18 percent for probationers to about 0.28 percent for state prisoners. BAC levels of 0.05 percent or higher were estimated by 90 percent of inmates convicted of murder and sexual assault who reported drinking at the time of their crimes, by 86 percent of those convicted of robbery, and by 78 percent of those convicted of assault (Greenfield 1998).

This BAC data cannot be taken as proof that drinking leads to violent crime. Other variables, such as poverty, family problems, antisocial personality disorder, or genetic predisposition, may cause both drinking and criminal acts. Lipsey and colleagues (1997) conducted a meta-analytical review of 129 studies, including general population surveys, samples of criminals, and samples of clinical populations (e.g., family therapy clients, and alcohol or drug treatments clients). They found that studies which were better controlled for other factors that might influence violence and aggression showed lower associations between alcohol and crime. Moreover, BAC data cannot be interpreted as indicating causality because intoxication may increase the chances that an offender will be apprehended at the scene, leading to overestimates of average offender BAC values.

### Longitudinal Studies

Evidence from longitudinal studies of young people casts doubt on a simple causal association between alcohol consumption and aggression or violence (White et al. 1993; White 1997; Zhang et al. 1997). These studies commonly show that alcohol abuse and violent behavior are associated later in life. However, later aggressive behavior and alcohol abuse were more strongly associated with early aggressive behavior than with early alcohol use. This suggests that early aggression may lead to later aggression and alcohol abuse, thus accounting for the correlation between the latter two variables. Further, a person’s level of aggressiveness is relatively stable from childhood to young adulthood, and young people may behave aggressively whether they drink or not. Reviews of longitudinal studies (e.g., White 1997) commonly conclude that the relationship between alcohol and aggression among young people is caused by a third factor or set of factors such as family variables (e.g., childhood neglect and abuse) or genetic and temperament traits (e.g., impulsivity, hyperactivity).

## Alcohol’s Effects on the Family

In addition to the harm that alcohol consumption causes for drinkers themselves, family members—especially spouses and children—are likely to be harmed as well (Maffli 2001). Both spouses and children can be victims of alcohol-related violence, and children can also suffer medical and social problems that may persist into adulthood.

### Effects on Children

Parental alcohol use can influence a child by genetic or prenatal means or by its impact on the child’s environment (Schuckit 1994; Schuckit and Smith 1996; Windle 1997). Fetal alcohol syndrome (FAS) is one of the most common direct consequences of parental alcohol use. Three to 10 out of every 10,000 babies born in the United States have been estimated to have fetal alcohol syndrome, but some sources estimate up to 30 or more FAS cases per 10,000 (Larkby and Day 1997; U.S. Department of Health and Human Services 2000). An Institute of Medicine report estimated that 0.5 to 3 cases of FAS occur per 1,000 births (Stratton et al. 1996).

Child abuse can be another direct consequence of parental alcohol use. English and colleagues (1995) concluded, based on evidence from case studies, that alcohol use is a cause of child abuse in an estimated 16 percent of cases. Criteria for their assessment included “reported misuse of alcohol in the family,” “intoxication reported by perpetrator,” or “history of alcoholism.” These authors could not identify any study that linked the risk of child abuse to particular levels of alcohol intake, however, nor could the authors of a followup report (Ridolfo and Stevenson 2001).

Parental drinking can affect the environment in which a child grows up by playing a part in:

- Acute and chronic financial strain— for example, because of excessive unemployment (Marmot et al. 1993)
- Poor parenting—for example, a coercive interaction style, inconsistent reinforcement of good behavior, or unclear behavioral expectations by the parents (Jacob and Johnson 1997)
- Marital conflicts and family breakup (Eurocare 1998; Leonard and Rothbard 1999)
- Creating expectations—that is, teaching the child to expect specific results from drinking (Sher et al. 1996).

These factors, and any interaction between them, may have a powerful impact on the life of a growing child.

### Partner Violence

Although most of the explanations of the link between alcohol and violence apply to partner as well as nonpartner violence, there appear to be some differences between the two types of events. Alcohol is commonly involved in a higher percentage of assaults between partners than in violent incidents between other people (Greenfeld 1998). The fact that two partners live together creates more opportunities for violent encounters and may account for the higher association between alcohol use and partner violence compared with nonpartner violence according to NCVS, 65 percent of the victims of spousal violence reported that alcohol was involved in the crimes against them—a larger percentage of involvement than reported by victims of any other type of crime (Greenfield 1998).

Another difference between partner and nonpartner violence is that the association between alcohol and partner violence appears to be less influenced by confounding variables than is the association between alcohol and nonpartner violence (Lipsey et al. 1997).

Third, the intoxication–victimization hypothesis—that is, that women under the influence of alcohol are more likely to become targets—is less clear in husband-to-wife violence compared with nonpartner violence (U.S. Department of Health and Human Services 2000; Chermack et al. 2001). Some, but not all, investigators have found that alcohol-related assaults were more likely when both partners were heavy drinkers (Kaufman Kantor and Asdigian 1997). Leonard and Roberts (1998), in one of the rare experiments on couples, found that alcohol consumption by both the wife and the husband increased negative interactions during a problem-solving task. It has often been found that males’ expectations result in an increased feeling of dominance under the influence of alcohol (Kaufman Kantor and Asdigian 1997). Thus, because problem-solving ability is impaired under the influence, attempts at problem solving together with the feeling of dominance may result in aggressive acts.

Other analyses indicate that the victim’s drinking is less likely to facilitate aggression by a partner than by a stranger (Kaufman Kantor and Asdigian 1997). A stranger may be more likely to gain access to a victim if the victim is intoxicated (Testa and Parks 1996).

The main problem with research on marital violence, as stated by Leonard and Roberts (1998), is that drinking patterns and usual consumption have rarely been adequately measured, and research in this area tends to use imprecise and sometimes impressionistic labels of problem drinkers or alcoholics. Thus it is not known whether problem drinking refers to a consistently heavy drinking pattern or to sporadic heavy drinking episodes by usually low-to-moderate drinkers. In one of the rare studies including a measure of heavy drinking episodes, Kaufman Kantor and Straus (1987) analyzed typical frequency and amount of drinking and found that the percentage of violent husbands in each drinking group increased linearly from abstainers to low drinkers to binge drinkers.^4^ This percentage was higher among binge drinkers than among regularly heavy drinkers.^5^ These authors also found that 19.4 percent in the low-drinking group but 48.4 percent in the bingeing group were drinking at the time of the violent event. Thus this study supports the idea that spousal violence is more likely not only when a partner is alcohol dependent or a problem drinker, but also when the partner is an infrequent drinker who occasionally drinks heavily.

## Methodological Considerations

Although drinking is known to be involved in many negative outcomes, research has yielded little substantial evidence on the mechanisms by which alcohol might cause those outcomes or even on levels of alcohol involvement. Many studies of social harm do not take into account the complexity of interactions among causative factors, transforming these interactions among factors into hypotheses that can be tested using appropriate statistical methodology. If theory postulates that violence is a consequence of complex interactions in a defined context within a cultural setting, it is meaningless to measure only the BAC of one person who has beaten another.

Specifically, research in this area would benefit from improvement in the use of control groups, in consideration of the chronological order of events, and in measurement of alcohol consumption and outcomes (Collins and Messerschmidt 1993; Gmel and Gutjahr 2001).

### Control Groups

Drawing conclusions about any connection between alcohol consumption and specific negative outcomes implies variability—for example, if the amount consumed goes up, the rate of injury goes up as well. The usual means of addressing variability is by comparing cases (i.e., people involved in an incident) with control subjects (i.e., uninvolved people). Looking at the BAC levels of people involved in a type of incident is not sufficient, as it is does not take variability into account.

Control subjects can be incorporated statistically or by using a case control design. It is critical to choose controls that are as similar as possible to cases (see Gmel and Gutjahr 2001): Cases of violence in bars should not be compared with controls of violence at home, and cases of violent injuries should not be compared with controls of nonviolent injuries.

At-scene studies are among the best case control designs. When conducted near the time as well as at the location of an event (e.g., in bars, on the road), they permit the collection of cases and control subjects directly, thus holding context and to a certain degree even situation constant. The most important advantage of at-scene, near-event studies is straightforward: Control subjects interviewed at the scene have the same situational factors as the cases (e.g., noise, weather conditions). The drawbacks are that such a design is geographically limited, a system for alerting researchers must be established, and the research team must arrive immediately after the event. An alert system is possible when the research is conducted in clearly defined places such as bars or workplaces, especially in cases where it is possible to determine days of the week or times of day when the risk of violence is greatest. At-scene studies of domestic violence are difficult because such incidents tend to occur in private homes.

Although there are advantages to conducting research at the scene and near the time of an event, research that relaxes these strict conditions would nevertheless result in an improvement of currently existing knowledge. For example, control subjects can be sampled at the scene but at another time (that is, independent of cases) if situational factors, such as time of day, noise level, and weather conditions, are well represented. For example, workers or visitors in bars can be interviewed during the week even when incidents have not occurred. At-scene designs have been approximated in roadside surveys (Hurst et al. 1994; Krüger 1995) in which random BAC testing was carried out in the same geographical area and over the same time span on drivers who were not involved in crashes and then compared with drivers who were involved in crashes. These at-scene designs should be possible when measuring other social consequences as well.

### Chronological Ordering

On the basis of much of the research in this area investigators are unable to draw conclusions about chronological order of events—for example, did a change in work conditions precede heavy alcohol use and an increase in work absences? Caution is needed when determining the order in which drinking problems, social consequences, and other variables (such as mental disorders) occur. To clarify alcohol’s role, longitudinal designs are essential in order to determine when alcohol consumption entered the sequence of events and to monitor whether drinking exacerbated or reduced consequences in people with comorbid disorders. Because many outcomes repeat over time, such a longitudinal study would benefit from including multiple measurement points, rather than only two (i.e., baseline and outcome), and adequate statistical techniques to examine causality (Robins et al. 1999).

### Measurement of Alcohol Consumption

Better measurement of alcohol consumption is critical to studies of harmful use. The preferred methods are determining BAC from blood samples or measuring breath alcohol concentrations using appropriate devices. In the absence of objective measures such as these, consumption can be estimated using self-reports (Greenfeld 1998) or observation of signs of intoxication (e.g., McClelland and Teplin 2001), bearing in mind, however, the subjectivity and other limitations of these approaches.

In addition to using the best measure of consumption at the time of an event such as a violent act, research would benefit from measurement of drinkers’ usual patterns of consumption, and more of a focus on light-to-moderate drinkers as well as heavy drinkers. Measuring usual patterns of drinking in addition to drinking contemporaneous with a specific event would allow researchers to address the question of whether negative outcomes are related to people’s usual drinking patterns or to isolated episodes of heavy drinking. Looking at light-to-moderate drinkers as well as heavy drinkers would make it possible to compare the occurrence of negative outcomes for a wider range of drinking patterns.

The difficult task of obtaining information on a person’s usual consumption and his or her drinking at the time of an event can probably be best accomplished in designs that permit linkage to records such as police reports and court documents. Case control studies also allow researchers to determine usual and contemporaneous consumption by asking people involved in nonfatal incidents about their usual drinking patterns, and by eliciting collateral reports on consumption related to fatal incidents.

### Measurement of Outcomes

Inadequate measurement of outcomes seems to characterize much research in the area of harmful use. Outcomes are defined and measured differently in different studies. Aggression may mean the pressing of a key labeled “shock” in a sterile experimental room or a verbal insult or a hostile thought about another person. Clearly, there is no reason why all these actions should have the same relation to alcohol. There is also no clear common metric with which to compare these outcomes. In medical research, measures such as YLLs (years of life lost) and DALYs (disability adjusted life years) can be used to make comparisons, for example, between diseases as different as the flu and liver cancer. Similar metrics are needed for nonmedical outcomes such as the social consequences of alcohol misuse.

## Conclusions

With the focus on specific negative consequences (workplace absenteeism, unintentional injuries, aggression and violence, spouse abuse), the present study examined knowledge of harmful alcohol use as defined by the alcohol abuse criteria in DSM–IV. In general, it was concluded that many—if not most—studies on these topics show methodological weaknesses. Therefore, the evidence for the effects of alcohol consumption on the examined consequences is sometimes weak, particularly as regards differential effects of different drinking patterns. The following conclusions can be drawn.

### Alcohol Consumption and the Workplace

Although moderate alcohol consumption may favorably affect productivity on the job, heavy drinking and alcohol abuse and dependence lower productivity. The evidence for an association specifically between workplace absenteeism and the full range of alcohol consumption (i.e., from abstention to heavy drinking), however, is inconclusive. Using a combination of retrospective assessment, self-reporting, and questions that attribute problems to alcohol consumption may overestimate the effect of drinking on absentee rates. On the other hand, if respondents tend to deny or cover up drinking problems, using self-reports may lead to an underestimate of effects. Confounding variables such as other job and personality characteristics may account for the inconclusiveness of research in this area. Longitudinal research and other measures of consequences rather than drinkers’ self-reports could perhaps best elucidate the complex causal webs linking alcohol consumption, absenteeism, and other variables.

### Alcohol Consumption and Unintentional Injuries

The research evidence indicates a high level of alcohol involvement in all types of unintentional injuries. The number of drinks consumed per occasion, especially when indicated by BAC, is strongly associated with the occurrence of injuries, independent of the usual frequency and quantity of alcohol consumed. Drinking may be less associated with workplace injuries for various reasons, but appears to play a role in causing falls, the second most common form of unintentional injury.

### Alcohol Consumption and Violence

Although alcohol is clearly associated with violent behavior, research evidence has not established that drinking causes violent acts. Experimental studies do not support the idea that alcohol acts pharmacologically to weaken people’s inhibitions against acting aggressively. These studies have suggested, however, that factors such as perception of threat or frustration may be more important in determining behavior than people’s expectations about how alcohol will affect them. Lastly, experimental data have yet to confirm or refute hypotheses that drinking changes people’s ability to attend to environmental cues, or that culture and social context influence the likelihood that drinkers will act aggressively.

Observational and longitudinal research supports an association between drinking and aggressive behavior. Reports from victims and convicted offenders indicate a considerable degree of alcohol involvement in violent crime, but do not prove causality. Other social, personality, and genetic factors may cause both drinking and violent behavior. Reviews of both longitudinal and other types of observational research commonly conclude that other family, temperament, or genetic factors account for the relationship between alcohol and aggression in young people.

### Alcohol Consumption and the Family

Alcohol misuse is associated with numerous negative consequences for the drinker’s partner and children. Maternal drinking during pregnancy can result in fetal alcohol syndrome in children, and parental drinking is correlated with child abuse and impacts a child’s environment in many social, psychological, and economic ways. Regarding partner violence, research evidence

- Indicates that it is more strongly associated with heavy drinking, whether usual or occasional, than is nonpartner violence
- Conflicts as to whether drinking by the victim makes violent acts by a partner more likely
- Suggests that alcohol consumption’s stronger association with partner violence than with nonpartner violence may be a matter of access, with partners having more contact and thus more opportunity for violent encounters.

## Figures and Tables

**Table 1:** Estimated Percentages of Non-Traffic-Related Unintentional Injury Deaths in Which Alcohol Was Involved, by Cause of Injury (United States, 1999)

Cause of Death	Positive*	Intoxicated**
**Unintentional injury**	38.5	31.0
Burn/fire	37.9	41.9
Cold/hyphothermia	90.0	40.9
Drowning	49.2	34.2
Fall	63.3	32.2
Gunshot	48.7	20.5
Poisoning by solid, liquid, or gas	26.6	29.3
**Motor vehicle crash**	39.7	32.8

* Blood alcohol concentration > 0 mg/dL.

** Blood alcohol concentration ≈ 100 mg/dL.

NOTE: Percentages of “positive” and “intoxicated” cannot be compared directly for a given type of injury because percentages were calculated separately based on meta-analysis of different studies. For example, 37.9 percent of burn/fire injuries were “positive,” but 41.9 percent were “intoxicated.”

SOURCE: Derived from a meta-analysis by Smith et al. (1999).

**Table 2:** Estimated Percentages of Violent Deaths in Which Alcohol Was Involved, by Cause of Injury (United States, 1999)

Cause of Death	Positive*	Intoxicated**
Total Homicides	47.1	31.5
Asphyxiation, hanging, strangulation, or suffocation	29.7	16.0
Burn/fire	36.4	18.2
Drowning	50.0	50.0
Beating, bludgeoning, using fists, feet, or blunt object	40.7	24.9
Gunshot	38.9	30.6
Stabbing, cutting, or piercing	57.0	43.0

* Blood alcohol concentration > 0 mg/dL.

** Blood alcohol concentration ≈ 100 mg/dL.

NOTE: Percentages of “positive” and “intoxicated” cannot be compared directly for a given type of injury because percentages were calculated separately based on meta-analysis of different studies. For example, 38.9 percent of gunshot injuries were “positive,” but 30.6 percent were “intoxicated.”

SOURCE: Derived from a meta-analysis by Smith et al. (1999).

## Footnotes

1 Alcohol consumption here refers to a person’s rate of drinking throughout his or her lifetime, not acute, situation-specific drinking.

2 Almost half of unintentional injury deaths in the United States in 1999 were caused by car crashes (Hoyert et al. 2001).

3 Drinking after the injury or a time lapse between injury and BAC reading, which is often a factor in Emergency Room studies (Voas 1993), can bias BAC data.

4 The binge drinking group in this study was defined as drinking with a frequency of twice a week or less often, and a quantity of five or more drinks per occasion.

5 This group (called the “high” drinking group in the study) was defined by a drinking frequency of at least three to four times a week and a usual quantity of at least three drinks a day.
